# Patterns of pain location in music students: a cluster analysis

**DOI:** 10.1186/s12891-021-04046-6

**Published:** 2021-02-14

**Authors:** Cinzia Cruder, Marco Barbero, Emiliano Soldini, Nigel Gleeson

**Affiliations:** 1 Rehabilitation Research Laboratory 2rLab, Department of Business Economics, Health and Social Care, University of Applied Sciences and Arts of Southern Switzerland, Manno/Landquart, Switzerland; 2 Centre for Health, Activity and Rehabilitation Research, Queen Margaret University, Edinburgh, UK; 3 Department of Research and Development, Conservatory of Southern Switzerland, Lugano, Switzerland; 4 Research Methodology Competence Centre, Department of Business, Health and Social Care, University of Applied Sciences and Arts of Southern Switzerland (SUPSI), Manno, Switzerland

**Keywords:** Music students, Pain location, Musculoskeletal, Cluster analysis

## Abstract

**Background:**

According to existing literature, musicians experience high rates of musculoskeletal (MSK) disorders involving different anatomical areas. The aim of the study was to identify patterns of pain location in a sample of music students enrolled in different pan-European music institutions. A further goal was to explore the association between the identified pain patterns and students’ characteristics.

**Methods:**

A total of 340 music students (mean age 23.3 years, 66.2% female) with current MSK pain completed a web-based questionnaire including both background information (i.e. lifestyle and physical activity, practice habits) and clinical features (i.e. pain characteristics, disability, pain self-efficacy, psychological distress, perfectionism and fatigue).

**Results:**

Five patterns of pain location were identified by hierarchical cluster analysis: wrist pain (WP) representing 22.6% of the total sample, widespread pain (WSP) (16.9%), right shoulder pain (RSP) (18.5%), both shoulders pain – left concentrated (LSP) (23.2%), neck and back pain (NBP) (18.8%). Amongst the identified patterns of pain location, bivariate analysis identified the WSP cluster as containing the largest number of associated variables. Participants in this cluster reported a higher percentage of women (*p* < .05), a higher perceived exertion (*p* < .01) and psychological distress (*p* < .001), as well as a lower level of self-efficacy (*p* < .01). Similarly, a higher percentage of participants included in the WSP cluster perceived their musical activity as the main cause of their MSK pain (*p* < .01). Additionally, a higher level of disability in relation to playing-related activity was reported by participants included in the WP and WSP clusters (*p* < .001). The RSP cluster was characterised by a higher percentage of participants playing an instrument in a neutral position (*p* < .001) and lower levels of socially prescribed perfectionism (*p* < .01). A higher percentage of participants playing an instrument with both arms elevated in the left quadrant position were included in the LSP cluster and a higher percentage of singers were included in the NBP cluster (*p* < .001).

**Conclusions:**

Five distinct patterns of pain location were identified and their associations with the students’ characteristics were explored. These findings may be helpful in the exploration of different aetiologies of MSK pain among musicians and in the development of targeted preventive strategies and treatments.

## Introduction

Musculoskeletal (MSK) disorders are common among musicians, with a point prevalence among professionals and music academy students oscillating between 9 and 68%, 12-month prevalence between 41 to 93%, and lifetime prevalence between 62 and 93% [1], with the possibility of tracking of mechanistic effects. Focusing specifically on music academy students, Kok et al. [2] reported a point prevalence of 63% of disorders of the MSK system and a 12-month prevalence of 89%. Similarly, in a recent study involving several universities of music in Europe, 65% of music students reported painful MSK disorders in the past 12 months [3].

Prevalence of MSK disorders by anatomical location is conflicted within the medical literature and hampered by heterogeneity of reporting symptoms. Several cross-sectional studies on musicians’ health reported the neck and shoulders as well as the back as being the most frequently affected regions [4–10]. In a recent study among music students from different U.S. college music programs [9], the most frequently affected locations overall were both upper and low back, fingers, left shoulder and throat. Approximately 75% of students reported that pain affected their ability to play or to sing, with approximately 40% of those with pain experiencing a certain degree of disability. On the other hand, a systematic review has reported the neck and shoulders as being the most affected anatomic regions amongst musicians [11], with left and right sides of the body influenced similarly.

Heterogeneity in reporting musicians’ disorders may be attributed to using different methods of assessment [11, 12], even though the Nordic Musculoskeletal Questionnaire (NMQ) remains the most commonly used questionnaire the analysis of MSK symptoms in the literature [13]. The extent of variability between the anatomical regions included within the literature, is illustrated by reports recording from four (i.e. neck, one or both shoulders, fingers) up to 32 locations [1], which inevitably limits generalisations and meaningful comparisons. Heterogeneous reporting of symptoms may also hamper the characterisation of the interactions with clinical features and their association with treatment outcomes.

An adjunct approach for defining empirically derived subgroups of musicians according to the anatomical distribution of their MSK pain and building upon contemporary evidence is needed. This could help in the exploration of different aetiologies and in the subsequent development of tailored treatment strategies to address MSK disorders among musicians. The multivariate statistical procedure of cluster analysis has provenance in medicine, psychology, sociology and marketing for identifying homogeneous groups from selected characteristics [14, 15], but has not yet been deployed amongst musicians.

This study aimed to identify distinct patterns of pain location in a sample of music students enrolled in different pan-European music institutions using cluster analysis. Additionally, the associations between the resulting patterns and their demographic, health-status and musical instrument-playing characteristics were explored.

## Materials and methods

This study features a sample of music students included in the Risk of Music Students (RISMUS) study, a longitudinal investigation identifying factors associated with increased risk of playing-related musculoskeletal disorders in music students. RISMUS was conducted between November 2018 and January 2020 to obtain self-reported data from a large population of music students of different Pan-European university schools of music. It featured 6-month and 12-month follow-ups characterising the time course of developing playing-related musculoskeletal disorders (PRMDs) at different stages of musical training. The study’s rationale, protocol [16] and overviewed data from all participating music students [3] has been described previously. This article consists of a secondary analysis of clinical features, focusing only on students reporting current MSK pain at baseline. All participants received written information prior to the study and signed informed consent. The Research Ethics Committee of Queen Margaret University of Edinburgh (REP 0177) provided ethical approval, with procedural oversight according to the 1964 Helsinki declaration and its later amendments.

### Participants

Participants were drawn from 850 music students enrolled in pan-European universities and the RISMUS longitudinal study. Inclusion required self-reported current MSK pain [16] (i.e. occurring within 1 month prior to survey’ completion), aged > 18 years old, and be a Pre-college and/or university-level student playing a musical instrument commonly used in classical music as a main subject. Exclusion criteria comprised participants having suffered severe, disabling neurological and/or rheumatic (e.g. fibromyalgia syndrome, rheumatoid arthritis, focal dystonia) and/or psychological (e.g. diagnosed severe borderline personality disorders) conditions in the past 12 months.

Eligible students received relevant information, consent form and a link to a web-based questionnaire by e-mail, with distribution and recruitment overseen by registrars of the music universities and conservatories [3]. A reminder e-mail was sent 3 weeks after the first e-mail.

### Materials

The web-based questionnaire included three sections:

#### Background information

General background questions elicited information on participants’ age, gender, self-reported height and weight, nationality, smoking status and sleeping habits, as well as practice habits (i.e. main instrument, academic level, average time playing per week and years of experience, the perceived exertion after 45 min of practice [0 “very low” to 10 “very high”], preparatory exercises and breaks during practice), health history (i.e. current medication, any neurological and/or rheumatic and/or psychological disorders in the past 12 months and any surgeries/accidents of the upper limbs and/or the spine in the past 12 months), the perceived health status (Self-Rated Health (SRH) [17] and the presence of MSK pain.

#### Description of current MSK pain

Further questions elicited information on (a) the duration and the type; (b) Playing-related Musculoskeletal Disorders (PRMDs), according to Zaza et al. [18] and if the perceived cause of their pain is attributed to the musical activity; (c) intensity assessed with a Visual Analogue Scale (VAS); (d) disability assessed with the Performing Arts Section of the Quick Disabilities of the Arm, Shoulder and Hand Outcome Measure (PAS –QuickDASH) [19] and the Pain Disability Index (PDI) [20–22]; (e) self-efficacy assessed with the 2-item short form of the Pain Self-efficacy Questionnaire (PSEQ-2) [23]; (f) location assessed with the Nordic Musculoskeletal Questionnaire (NMQ) [24].

#### Physical and psychological characteristics

This last section included the International Physical Activity Questionnaire – short form (IPAQ-SF) [25] for the assessment of physical activity participation level; the Kessler Psychological Distress Scale (K10) [26] for the assessment of anxiety and stress; the Multidimensional Perfectionism Scale – short form (HFMPS-SF) for the assessment of perfectionism [27–29]; The Chalder Fatigue Scale (CFQ 11) for the assessment of fatigue [30].

Participants were allocated into six categories according to the classification of Kok et al. [31], which in turn had been based on the study by Nyman et al. [32]. This classification focused on elevation of the arm while playing (i.e. ≥40° abduction and/or ≥ 40° forward flexion) as a risk factor for MSK pain and had been further adapted from a previous study [3]. The latter included two additional categories: “both arms elevated in a frontal position” and “both arms elevated in the left quadrant position” and the category of “singers”, due to the specific characteristics of their musical practice [4].

### Statistical analysis

Descriptive statistics were used to summarise and present the data. For categorical variables, absolute and relative frequency distributions were presented, while continuous variables were described using medians and ranges.

Binary variables (pain; no pain) related to the location of current MSK pain were clustered according to the Balanced Iterative Reducing and Clustering using Hierarchies (BIRCH) algorithm [33]. A first phase produced subgroups of observations based on log-likelihood distance, with homogeneously clustering through a hierarchical agglomerative clustering procedure in a second phase. The final number of patterns of pain location was empirically determined by the Bayes Information Criterion (BIC) and the overall goodness-of-fit of the clustering procedure was assessed using the average silhouette coefficient.

Finally, bivariate statistical tests were used to identify the associations between patterns of pain location and the demographic variables, those associated with self-reported health-related status and with the playing of musical instruments, as well as variables associated with MSK pain (i.e. duration, pattern, intensity, PRMDs, cause attributed to the musical activity, disability and self-efficacy). Specifically, the chi-square test was used to analyse the association between the identified pain patterns and the categorical variables, while the Kruskal-Wallis test, besides accommodating the potential for non-normality of the distributions, was used to identify the association with continuous variables.

## Results

A cohort of 340 participants (40% of 850 RISMUS baseline participants) were retained for the present study.

### Descriptive statistics

Cohort’ demographic variables (Table 1), variables associated with self-reported health-related status (Table 2), variables associated with the playing of musical instruments (Table 3) and variables of MSK pain (Table 4) are shown below.

**Table 1:** Descriptive statistics of demographic variables

Variable	n	%
Gender (*n* = 340)		
Woman	225	66.2%
Man	112	32.9%
Other	3	0.9%
Age (*n* = 340)		
median	22	
range	18–45	
Nationality (region)^a^ (*n* = 340)		
South Europe	184	54.1%
West Europe	94	27.7%
North Europe	28	8.2%
East Europe	17	5.0%
Other	17	5.0%
Academic level (*n* = 340)		
Pre college	32	9.4%
Bachelor 1&2	51	15.0%
Bachelor 3&4	69	20.3%
Master 1&2	55	16.2%
Master 3&4	73	21.5%
Gap year experience/continuing education	60	17.6%

^a^This classification was made according to United Nations, S. D. Standard Country or Area Codes for Statistical Use, Series M, No. 49 (M49) <https://unstats.un.org/unsd/methodology/m49/> (1999)

**Table 2:** Descriptive statistics of variables associated with health-related status

Variable	n	%
BMI in kg·m^−2^ (*n* = 332)		
Median	21.5	
Range	15.3–35.9	
Perceived health [SRH] (*n* = 340)		
Excellent	16	4.7%
Very good	74	21.8%
Good	174	51.2%
Fair	67	19.7%
Poor	9	2.7%
Hours of sleep (*n* = 340)		
Median	7	
Range	4–9	
Smoking (*n* = 340)		
Yes	63	18.5%
No	277	81.5%
Medications (*n* = 340)		
Nothing	273	80.3%
Supplement/contraceptive	29	8.5%
Medicine	38	11.2%
Physical activity status [IPAQ-SF score] (*n* = 337)		
Low	60	17.8%
Moderate	178	52.8%
High	99	29.4%
Psychological distress [K10 score] (*n* = 337)		
Median	21	
Range	10–45	
Perfectionism [HFMPS-SF score]		
SO sub-scale score (*n* = 329)		
Median	26	
Range	9–35	
OO sub-scale score (*n* = 334)		
Median	19	
Range	7–35	
SP sub-scale score (*n* = 333)		
Median	18	
Range	7–35	
Fatigue [CFQ 11 score] (*n* = 332)		
Median	15	
Range	0–33	

*BMI* Body Mass Index, *SRH* Self-rated health, *IPAQ-SF* International Physical Activity Questionnaire - short form, *K10* Kessler Psychological Distress Scale, *HFMPS-SF* Multidimensional Perfectionism Scale - short form, *SO* Self-oriented, *OO* Other-oriented, *SP* Socially prescribed, *CFQ 11* Chalder Fatigue Scale

**Table 3:** Descriptive statistics of variables associated with the playing of musical instruments

Variable	n	%
Instrument [classification] (*n* = 340)		
Elevated both frontal	28	8.2%
Elevated both left	65	19.1%
Elevated left	28	8.2%
Elevated right	50	14.7%
Neutral	132	38.8%
Voice	37	11.0%
Years of practice (*n* = 340)		
Median	13	
Range	6–34	
Hours of practice per day (*n* = 340)		
Median	3	
Range	3–8	
Perceived exertion after 45 min of practice without breaks (*n* = 340)		
Median	5	
Range	0–10	
Preparatory exercises (*n* = 340)		
Yes	149	43.8%
No	191	56.2%
Breaks during practice (*n* = 340)		
Yes	210	61.8%
No	130	38.2%

Elevated both frontal: Music students playing musical instruments with both arms elevated in a frontal position (i.e. harp, trombone, and trumpet); Elevated both left: Music students playing musical instruments with both arms elevated in the left quadrant position (i.e. viola, violin); Elevated left: Music students playing musical instruments with only the left arm elevated (i.e. cello, double bass); Elevated right: Music students playing musical instruments with only the right arm elevated (i.e. flute, guitar); Neutral: Music students playing musical instruments in a neutral position, without the elevation of arms (i.e. accordion, bassoon, clarinet, euphonium/tuba; French horn, harpsicord, oboe, organ, percussion, piano, recorder, saxophone)

**Table 4:** Variables of MSK pain

Variable	n	%
Type (*n* = 340)		
Continuous steady constant	112	32.9%
Rhythmic periodic intermittent	134	39.4%
Brief momentary transient	94	27.7%
Intensity [VAS] (*n* = 340)		
Median	4	
Range	1–10	
Disability [PAS-QuickDASH score] (*n* = 232)		
Median	31.3	
Range	0–100	
Disability [PDI score] (*n* = 265)		
Median	15	
Range	1–51	
Pain self-efficacy [PSEQ-2 score] (*n* = 340)		
Median	10	
Range	12–2	

*VAS* Visual Analogue Scale, *PRMD* Playing-Related Musculoskeletal Disorders, *PAS-QuickDASH* Performing Arts Section of the Quick Disabilities of the Arm, Shoulder and Hand Outcome Measure, *PDI* Pain Disability Index, *PSEQ-2* 2-item short form of the Pain Self-Efficacy Questionnaire

**Fig. 1:**
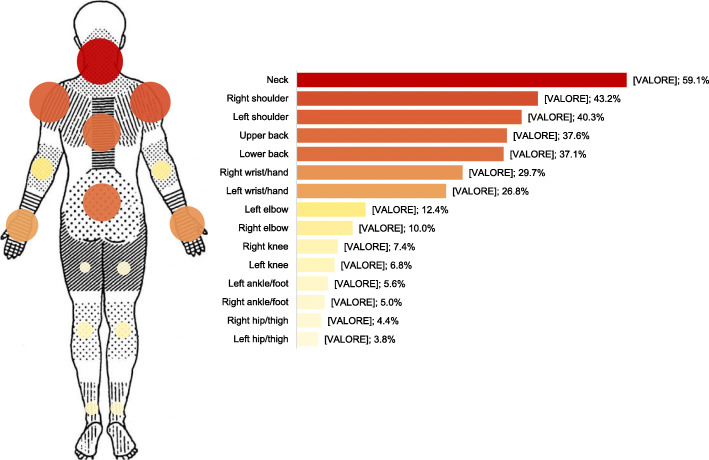
Distribution of MSK pain location among participants. The anatomical areas and the layout of the original Nordic Questionnaire [24] with the affected areas, as well as the graph with the number of participants who self-reported MSK pain in specific areas of the body, have been reported. Dark red represents the most frequently reported area throughout all participants

The location of MSK pain was assessed according to the 15 anatomical areas included within the Standardised Nordic Questionnaire [24]. The results indicated that the neck (59.1%) and shoulders (43.2% on the right and 40.3% on the left) areas, as well as the back (37.7% in the upper part and 37.1% in the lower part) were the most frequently affected areas throughout the participants (see Fig. 1). The percentages regarding the occurrence of MSK pain by location would seem to indicate no differences between the left and the right side.

**Fig. 2:**
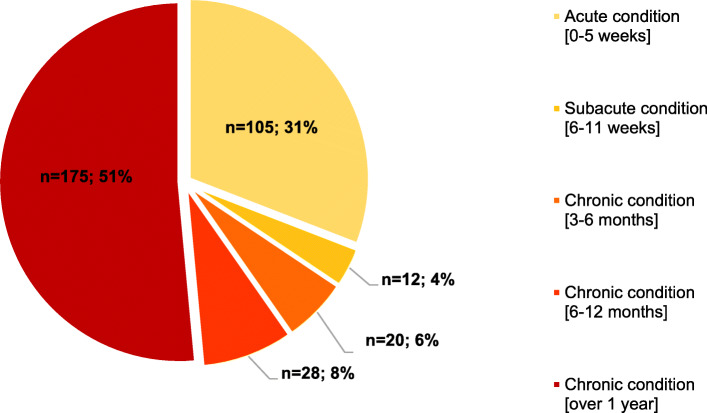
Distribution of different types of MSK pain according to the duration

About two-thirds of participants self-reported chronic MSK pain (for more than 3 months, according to the definition of “chronic pain” by the International Classification of Diseases (ICD) of the World Health Organization (WHO) [34], of whom 51.5% self-reported MSK pain for over a year (see Fig. 2).

**Fig. 3:**
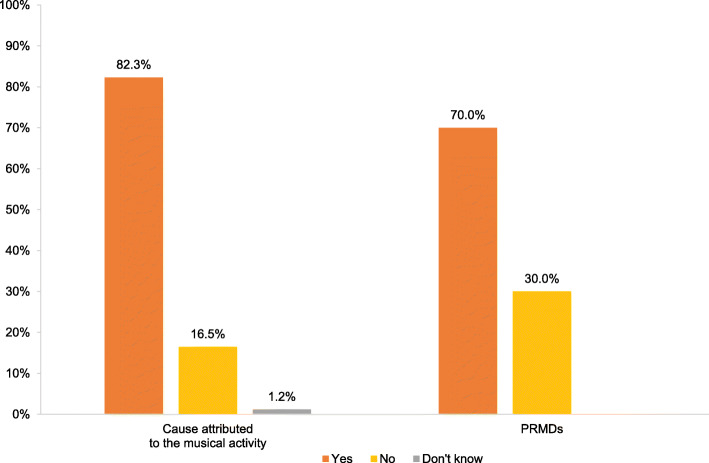
Distribution of participants reporting the musical activity as the perceived cause of their MSK pain and the prevalence of self-reported playing-related musculoskeletal disorders (PRMDs), according to the definition of Zaza et al. [18]

As seen in Fig. 3, a large proportion of participants perceived the musical activity to be causing their MSK pain (82.3%; Fig. 3). Finally, 70% of participants considered their MSK pain as a playing-related musculoskeletal disorder (PRMDs) and thus interfering with their playing ability, as defined by Zaza et al., 1998 [18].

### Cluster analysis

The BIRCH algorithm was applied to the 15 anatomical locations. Results showed that the six lower body variables (i.e. right and left hip, right and left knee, right and left ankle foot) offered sub-optimal clustering stability and robustness, probably due to their relatively low occurrence (less than 10%, see Fig. 1), which contaminated the efficiency of the process. Following elimination of the lower body variables, the BIRCH algorithm was reapplied to the nine upper body variables (i.e. neck, right shoulder, left shoulder, upper back, low back, right elbow, left elbow, right wrist/hand, left wrist/hand) and produced five distinct patterns of pain location (see Table 5). The average silhouette coefficient (i.e. 0.3) showed sufficient goodness-of-fit for the five patterns of pain location, with significant chi-square test scores (χ^2^_(df, 4)_ ranging from 20.96 to 270.65; *p* < 0.001) of associations between all candidate binary variables and the identified pain patterns, indicating their utility.

**Table 5:** Cluster analysis according to the location of MSK pain among participants

Patterns of pain locations						
Location	Total (*n* = 340)	WP (*n* = 77)	WSP (*n* = 57)	RSP (*n* = 63)	LSP (*n* = 79)	NBP (*n* = 64)
Neck	59%	39% ** ↓	98% *** ↑	51%	61%	55%
Right shoulder	43%	3% *** ↓	63% ** ↑	100% *** ↑	58% * ↑	0% *** ↓
Left shoulder	40%	10% *** ↓	84% *** ↑	3% *** ↓	100% *** ↑	0% *** ↓
Right elbow	10%	17%	23% ** ↑	11%	1% ** ↓	0% ** ↓
Left elbow	12%	14%	49% *** ↑	0% ** ↓	3% * ↓	2% * ↓
Right wrist/hand	30%	70% *** ↑	49% ** ↑	30%	0% *** ↓	0% *** ↓
Left wrist/hand	27%	60% *** ↑	77% *** ↑	2% *** ↓	0% *** ↓	0% *** ↓
Upper back	38%	22% ** ↓	56% * ↑	30%	37%	48%
Lower back	37%	29%	56% ** ↑	19% ** ↓	38%	37%

*** *p* < 0.001, ** *p* < 0.01, * *p* < 0.05

WP (*n* = 77): wrist pain (representing 22.6% of the total sample); WSP (*n* = 57): widespread pain (16.9%); RSP (*n* = 63): right shoulder pain (18.5%); LSP (*n* = 79): both shoulders pain – left concentrated (23.2%); NBP (*n* = 64): neck and back pain (18.8%). To facilitate the description and interpretation, cluster percentages significantly lower than the total sample percentages (according to the two-sided Z-test for proportions and considering a p-value of 5% as threshold for statistical significance) are followed by a downward-facing arrow (↓), while percentages significantly higher are followed by an upward-facing arrow (↑).

The distribution of pain location in the total sample and across the identified patterns is reported in Table 5. To facilitate the description and interpretation, cluster percentages significantly lower than the total sample percentages (according to the two-sided Z-test for proportions and considering a *p*-value of 5% as threshold for statistical significance) are followed by a downward-facing arrow (↓), while percentages significantly higher are followed by an upward-facing arrow (↑).

As can be seen in Table 5, participants in the wrist pain (i.e. WP) cluster, representing 22.6% of the total sample (*n* = 77), reported significantly more MSK pain in the wrist (70% in the right and 60% in the left; *p* < 0.001), when compared to the total sample (30 and 27%, respectively). By contrast, participants in the WP cluster reported less occurrence in the neck (39%; *p* < 0.01 vs. 59% of the total sample) and shoulders (3% in the right and 10% in the left; *p* < 0.001 vs. 43 and 40%, respectively of the total sample), as well in the upper back (22%; *p* < 0.01 vs. 38% of the total sample).

Participants included in the widespread pain (i.e. WSP) cluster reported significantly greater occurrence of more MSK pain in all locations (represented by reports from 23 to 98% of participants in the cluster; range: *p* < 0.05 to *p* < 0.001), compared to the MSK pain occurrence in the total sample (from 10 to 59%).

Participants in the right shoulder pain (i.e. RSP) cluster reported greater occurrence of MSK pain in the right shoulder (reported by 100% of cluster participants vs. 43% of the total sample; *p* < 0.001), but the opposite for the left shoulder (3% vs. 40% of the total sample; *p* < 0.001). Furthermore, RSP cluster membership was characterised by reported occurrence of MSK pain being significantly lower than in the total sample for the left elbow (0% vs. 12%, respectively; *p* < 0.01), left wrist/hand (2% vs. 27%, respectively; *p* < 0.001) and lower back (19% vs. 29%, respectively; *p* < 0.01).

Whereas belonging to the RSP cluster was characterised in particular by a significant difference in the occurrence of MSK pain between contralateral shoulders, participants in both shoulders – left concentrated pain (i.e. LSP) cluster reported greater occurrence of MSK pain in both left (100% vs. 40%, respectively; *p* < 0.001) and right shoulders (58% vs. 43%, respectively; *p* < 0.05), with a tendency in the left side, compared to the occurrence reported within the total sample.

Belonging to LSP cluster also reflected significantly lower occurrence of MSK pain compared to that amongst the total sample, in the elbows (right: 1% vs. 10%, respectively; *p* < 0.01; left: 3% vs. 11%, respectively; *p* < 0.05) and in both wrists (right: 0% vs. 30%, respectively; *p* < 0.001; left: 0% vs. 27%, respectively; *p* < 0.001), but these were anatomical locations in which the occurrence of MSK pain had been relatively low.

Finally, participants included in the neck and back pain (i.e. NBP) cluster reported MSK pain in the neck, upper back and lower back. However, the patterns of occurrence were similar statistically to those of the total sample.

### Bivariate analysis

Statistically significant relations with the identified pain patterns emerged for nine of the 29 variables considered (see Table 6).

**Table 6:** Results of the bivariate analysis

	Total (*n* = 340)	Patterns of pain location					Statistical test result
		WP	WSP	RSP	LSP	NBP	
		(*n* = 77)	(*n* = 57)	(*n* = 63)	(*n* = 79)	(*n* = 64)	
Gender							
Woman	66%	55%	79%	67%	71%	62%	χ^2^_(df, 8)_ = 18.05*
Man	33%	44%	18%	33%	29%	38%	
Other	1%	1%	3%	0%	0%	0%	
Perceived health [SRH]							
Excellent	5%	7%	5%	5%	5%	2%	χ^2^_(df, 16)_ = 28.30*
Very good	22%	27%	11%	33%	14%	23%	
Good	51%	45%	65%	40%	52%	56%	
Fair	20%	17%	22%	22%	24%	19%	
Poor	2%	4%	4%	0%	5%	0%	
Disability [PAS-QuickDASH score, 0–100]							
Median	31.3	37.5	37.5	34.4	25.0	25.0	χ^2^_(df, 4)_ = 22.20***
Self-efficacy [PSEQ-2 score, 12–0]							
Median	10.0	10.0	9.0	10.0	10.0	10.0	χ^2^_(df, 4)_ = 17.90**
Psychological distress [K10 score, 0–50]							
Median	21.0	21.0	25.0	20.0	22.0	21.0	χ^2^_(df, 4)_ = 22.60***
Perfectionism [HFMPS-SF – SP sub-scale score, 5–35]							
Median	18.0	17.0	20.0	16.5	20.0	19.0	χ^2^_(df, 4)_ = 13.57**
Instrument [classification]							
Elevated both frontal	8%	4%	18%	6%	9%	6%	χ^2^_(df, 20)_ = 49.53***
Elevated both left	19%	16%	26%	16%	29%	9%	
Elevated left	8%	12%	16%	5%	3%	6%	
Elevated right	15%	19%	10%	14%	7%	22%	
Neutral	39%	45%	25%	49%	37%	36%	
Voice	11%	4%	5%	10%	15%	21%	
Perceived exertion after 45 min of practice without breaks [0–10]							
Median	5.0	5.0	6.0	4.0	4.0	5.0	χ^2^_(df, 4)_ = 13.99**
Perceived cause attributed to the musical activity							
Yes	82%	87%	95%	87%	79%	66%	χ^2^_(df, 8)_ = 23.66**
No	17%	13%	5%	11%	20%	31%	
Don’t know	1%	0%	0%	2%	1%	3%	

*** *p* < 0.001, ** *p* < 0.01, * *p* < 0.05

For categorical variables, the total sample and cluster specific distributions of the variables considered (column percentages) have been reported, as well as the chi-square statistic and its statistical significance level. For continuous variables, the median and the range for each variable has been reported, as well as the chi-square statistic of the Kruskal-Wallis test and its statistical significance level

*WP* Wrist pain, *WSP* Widespread pain RSP, right shoulder pain, *LSP* Both shoulders pain - left concentrated, *NBP* Neck and back, *SRH* Self-rated health, *PAS-QuickDASH* Performing Arts Section of the Quick Disabilities of the Arm, Shoulder and Hand Outcome Measure, *PSEQ-2* 2-item short form of the Pain Self-Efficacy Questionnaire, *K10* Kessler Psychological Distress Scale, *HFMPS-SF* Multidimensional Perfectionism Scale - short form, *SP* Socially prescribed

Elevated both frontal: Music students playing musical instruments with both arms elevated in a frontal position (i.e. harp, trombone, and trumpet); Elevated both left: Music students playing musical instruments with both arms elevated in the left quadrant position (i.e. viola, violin); Elevated left: Music students playing musical instruments with only the left arm elevated (i.e. cello, double bass); Elevated right: Music students playing musical instruments with only the right arm elevated (i.e. flute, guitar); Neutral: Music students playing musical instruments in a neutral position, without the elevation of arms (i.e. accordion, bassoon, clarinet, euphonium/tuba; French horn, harpsicord, oboe, organ, percussion, piano, recorder, saxophone)

There was a lower percentage of women reporting MSK pain in the WP cluster (55%) and a higher percentage of women in the WSP cluster (79%). Participants included in the WSP cluster were more likely to report a good health status (65%) instead of a very good health status (11%), whereas a higher percentage of participants included in the RSP cluster reported a good (40%) and a very good health status (33%). In addition, a higher level of disability in the arm, shoulder and hand (PAS-QuickDASH score) was reported by participants included in the WP, WSP and RSP clusters, especially in the first two, where the median was in both cases 37.5. Conversely, the level of disability was much lower in the LSP and NBP clusters (the median was 25.0 in both clusters). Similarly, a higher level of perceived exertion after 45 min of practice without breaks was reported by participants of the WSP cluster, combined with lower scores of participants in the RSP and LSP clusters (the median was 4.0 in both clusters). Furthermore, a lower level of self-efficacy (PSEQ-2 score) (9.0, the lowest level among all clusters) and a higher level of psychological distress (K10 score) (25.0, the highest level among all clusters) were reported by participants included in the WSP cluster. In terms of perfectionism, a high degree of socially prescribed perfectionism was reported by participants included in the WSP (20.0) and NBP (20.0) clusters. Moreover, a higher percentage of participants playing an instrument with both arms elevated in a frontal position (18%) and a lower percentage of participants playing an instrument in a neutral position (25%) were included in the WSP cluster. On the other hand, a higher percentage of participants playing an instrument in a neutral position (49%) were included in the RSP cluster.

A higher percentage of participants playing an instrument with both arms elevated on the left side (29%) were included in the LSP cluster and a higher percentage of singers (21%) were included in the NBP cluster. Finally, a higher percentage of participants perceiving their musical activity as the cause of their MSK pain were included in the WSP cluster (95%) and a lower percentage of these participants were included in the NBP cluster (66%).

## Discussion

This study primarily focused on the exploration of patterns of pain location empirically identified in a cohort of music students enrolled in different pan-European music institutions.

Consistent with previous research [1, 4–9, 11, 35], our findings showed that the anatomical areas most affected by MSK pain among participants were the neck (59.1%) and shoulders (43.2% on the right and 40.3% on the left), as well as the back (37.7% in the upper part and 37.1% in the lower part).

Cluster analysis identified five homogeneous patterns of pain location amongst the 340 participants (see Table 5). The WP and WSP clusters were characterised by MSK pain in the wrists and by widespread pain (i.e. amongst all locations), respectively. MSK pain in the shoulders featured within both RSP and LSP clusters, with right shoulder only, and left shoulder emphasis amongst pain in both shoulders, respectively, as distinguishing characteristics. Participants included in the NBP cluster reported focal MSK pain in the neck, upper and lower back.

Amongst the identified patterns of pain location, the largest number of associated variables in the bivariate analysis emerged in the WSP cluster, which contained the most heterogeneous dispersal of location variables (i.e. widespread pain). This group identified a significant differential of MSK pain in women (79%) compared to men (18%; χ^2^_(df, 8)_ = 18.05 *p* < 0.05; Table 6), which is in line with previous studies [4, 7, 11]. Similarly, participants included in the WSP cluster were more likely to report a lower level of self-efficacy, where the median for the PSEQ-2 score was 9.0 in this group and 10.0 in the other clusters (see Table 6; χ^2^_(df, 4)_ = 17.9; *p* < 0.01) and a higher level of perceived exertion after 45 min of practice without breaks (see Table 6; χ^2^_(df, 4)_ = 13.99), where the median of the WSP cluster (i.e. 6.0) was the highest amongst all the groups. Interestingly, the WSP cluster’s median for musicians practicing in private was fairly high, considering the potential for increased ratings during performance [36].

Furthermore, widespread pain is often associated with psychological distress [37], and this was confirmed in our study. The median of the K10 score was significantly higher in the WSP cluster, presenting the highest figure among the clusters (see Table 6; χ^2^_(df, 4)_ = 22.6; *p* < 0.001). This finding is consistent with previous evidence of positive relationships between pain and depression [38, 39], tendencies to somatisation [38] and with anxiety [38, 39].

The aetiology of MSK pain in music students was further implicated within this study’s bivariate analyses, where the positive association between perceiving the musical activity as the cause of MSK pain and belonging to the WSP cluster (see Table 6; representing reports from 95% of participants in the WSP cluster vs. 82% of the total sample; χ^2^_(df, 8)_ = 23.66; *p* < 0.01; 95%), suggested a possible relationship between reporting widespread pain and a student’s playing activity. Nonetheless, the percentage of participants perceiving their musical activity as the main cause of their MSK pain was remarkably high among all groups (82.3%) and this is in line with previous research [36].

Additionally, the use of clustering also reveals substantial variation in the reporting of disability in regard to playing-related activities and the pattern of pain location. When compared to the total sample, a higher rate of disability in relation to playing-related activities was reported by participants included in the WP and WSP clusters and a much lower level was shown in the LSP and NBP clusters (see Table 6; χ^2^_(df, 4)_ = 22.2; *p* < 0.001). Indeed, PAS-QuickDASH scores of 37.5 recorded for both WP and WSP clusters, and 25.0 for both LSP and NBP clusters (Table 6) showed a wide range of scores compared to the median of the total sample (31.3). Overall, reported disability levels were high in comparison to other studies using this outcome measure among music students [40–42], professional orchestra musicians [6, 43] or among other populations [44]. Even though this difference could be attributed to the fact that participants included in our sample were all music students with current MSK pain, the mechanisms regarding the impact of MSK pain on their functional and the relevant implications on their playing ability deserve further exploration. For instance, future focal research involving selected played-instruments may reveal even more critical insights about MSK pain. Indeed, depending on the instrument played, musicians are exposed to rather uncomfortable, ergonomically incorrect positions and postures that often require static and prolonged use of the neck and shoulders as well as a repetitive use of the joints of the upper body, or a combination of both.

**Fig. 4:**
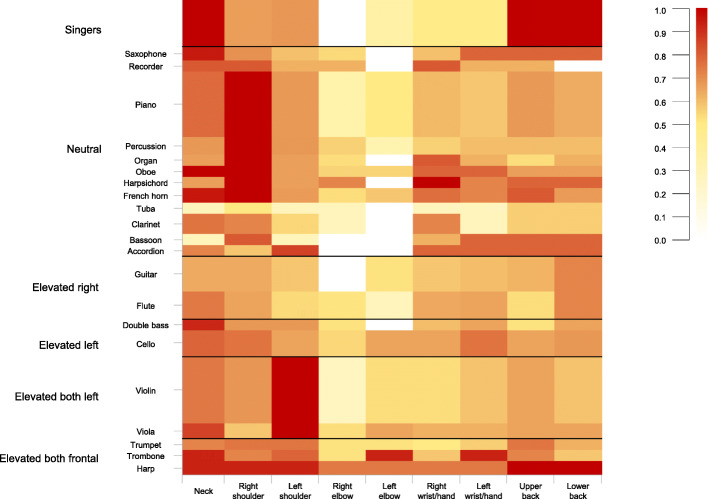
A heat map generated from the location of MSK pain data of the six groups, which have been divided according to the playing posture. Dark red represents the most frequently reported location. The vertical dimension of the six categories depends on the samples size of each group. Elevated both frontal (*n* = 28): Music students playing musical instruments with both arms elevated in a frontal position; Elevated both left (*n* = 65): Music students playing musical instruments with both arms elevated in the left quadrant position; Elevated left (*n* = 28): Music students playing musical instruments with only the left arm elevated; Elevated right (*n* = 50): Music students playing musical instruments with only the right arm elevated; Neutral (*n* = 132): Music students playing musical instruments in a neutral position, without the elevation of arms; Singers (*n* = 37)

In order to analyse differences in terms of MSK pain in different instrumental groups, the present study used the classification of risk associated with an elevated arm position [31, 32], which has been adapted according to a previous study [3]. Bivariate analysis regarding clusters’ membership and instruments’ classification revealed noteworthy associations (see Table 6; χ^2^_(df, 20)_ = 49.53; *p* < 0.001). Participants playing an instrument with “both arms elevated in a frontal position” were more likely to be included in the WSP cluster and participants playing an instrument with both arms elevated in the left quadrant position showed a statistically significant association with MSK pain in the shoulders – left concentrated, as expected. Moreover, participants playing instruments in a neutral position were more likely to be included in the RSP cluster and less in the WSP cluster. Ultimately, the category of singers, consistent with previous research [4], was more likely to be included in the NBP cluster and thus to report MSK pain more in the neck and in the back in comparison with the total sample. The latter would be due probably to the overuse of both the vocal tract and the standing position singers have to maintain for many hours during performance or rehearsals, especially in regard to the back [4]. These findings could be clearly observed also in the distribution of MSK pain in the various anatomic regions of the upper body among the six groups (see Fig. 4).

As might have been expected, the highest prevalence of MSK pain in the left shoulder was reported by participants playing with both arms elevated and in the left quadrant. Similarly, MSK pain reported for instruments played with both arms elevated in a frontal position covered almost the entire upper part of the body, especially the neck and shoulders, as well as the back for the harp players, and the left elbow, and wrist/hand for the trombone players. Asymmetry, which involves playing with one or both arms elevated, is a recognised issue in ergonomics for biomechanical risk assessment [32] and previous studies have demonstrated that working with elevated arms may lead to the degeneration of muscles and tendons, causing discomfort and distress [4, 32, 45–49].

Consequently, this study’s approach of statistically clustering musicians according to pain location patterns might have implications potentially for further research. The multivariate clustering approach based on homogeneity of patterning might be offering a more precise and empirical representation of the population’s burden and capable of providing distinctive information on trajectories of MSK pain among musicians, with a new interpretation that is different in its nature compared to antecedents within the literature view of evidence. This type of novel interpretation might reasonably form the basis for even more sophisticated and comprehensive long term-research to quantify the impact trajectories of patterns of MSK pain affecting musicians at specific anatomical sites, and the efficacy of standardised interventions for both primary and secondary prevention. For example, prophylactic strategies for the management of pain before its escalation to a chronic levels have been advocated [50], together with approaches offering greater insights into the exploration of different aetiologies and personal significance of pain amongst musicians.

Importantly, the technique, eliciting five empirically-derived pain patterns suggests that musicians with MSK pain should not be considered as a homogeneous group as this sub-optimal approach could be problematic and lead to inaccurate treatments. For example, principles of treatment specificity for optimal responses, might reasonably dictate that musicians with widespread pain should benefit most from a congruent array of treatment strategies. The latter might include appropriate multicomponent approaches emphasising integrated care for decreasing psychological distress and disability, as well as perceived exertion during practising. Future studies will be able to address whether the prevalence of MSK pain would be reduced when adopting such specific treatments when compared to contemporary practices.

### Limitations

There are limitations to be aware of when considering our findings. Firstly, although the location of MSK pain was determined according to a well-known and validated questionnaire - NMQ by Kuorinka et al. [24] - in order to obtain standardised results which could be compared to other groups [13], specific localised areas of the body such as fingers, were not contemplated as defined by other methods (e.g. anatomical regions according to the Margolis rating or according to pain drawings). This approach, in turn, was directly connected with the lack of a specific diagnosis for the MSK pain due to the self-reported nature of the study without any physical examination or objective measures and the use of a battery of questionnaires that were not validated with a web-based approach. Nonetheless, the self-reported web-based data was used in the best way possible to minimise potential heterogeneity amongst participants that had affected studies in the contemporary literature. Similarly, the use of validated measures in this context may contribute to and facilitate meta-analytical synthesis and further understanding of the study’s results. A more comprehensive investigation considering specific diagnosis may yield additional results capable of furthering our understanding of the relevance of studying MSK pain.

In addition, although all the available questionnaires’ official and validated translations (*inter alia* NMQ) were used as the present study involved many countries in Europe [3], the translation of some questionnaires was not publicly available and thus was performed by official interpreters. Consequently, they have not been submitted to a cross-cultural adaptation. However, as the sample size of the RISMUS project was relatively large, it might be feasible in the future to explore translated questionnaires’ relative stability in appearance and composition amongst different cultural adaptations.

Furthermore, limitations associated with the clustering analyses used in this study include the need for replication of the patterns of belonging observed in this study amongst other populations, for example within an even broader range of music students and amongst professional musicians or including external validation within independent populations. The current study reflects selected sub-sample’ responses of a relatively large (*n* = 997) group of music students from amongst those enrolled in 56 pan-European music institutions at baseline of the RISMUS project [3]. Nevertheless, altered heterogeneity amongst intra-individual and inter-individual characteristics associated with larger or different populations of musicians, might provoke incongruence with the findings of the exploratory models of this study. Future validation studies should evaluate the advantages of clustering as an adjunct to current diagnostic and treatment approaches. It is plausible that the latter approach might contribute to a wider understanding of musicians’ MSK pain as well as to the development of more effective treatment strategies for each kind of cluster.

## Conclusions

This study identified five homogeneous patterns of pain location for music students from different pan-European music institutions. Amongst the identified patterns of pain location, the largest number of associated variables in the bivariate analysis emerged in the WSP (i.e. widespread pain) cluster. Participants in this cluster reported a higher percentage of women, perceived exertion and psychological distress as well as a lower level of self-efficacy. Similarly, a higher percentage of participants included in the WSP cluster perceived their musical activity as the main cause of their MSK pain. Additionally, a higher level of disability in relation to playing-related activity was reported by participants included in the WP (i.e. wrist pain) and WSP clusters. The RSP cluster (i.e. right shoulder pain) was characterised with a higher percentage of participants playing an instrument in a neutral position and lower levels of socially prescribed perfectionism. A higher percentage of participants playing an instrument with both arms elevated on the left side were included in the LSP cluster (i.e. both shoulders pain – left concentrated) and a higher percentage of singers were included in the NBP cluster (i.e. neck and back pain).

This study contributes novel perspectives to the understanding and exploration of anatomical patterning of MSK pain within the community of music students. Our findings highlight the need for more effective evidence-based preventive strategies and tailor-made interventions for music students.

## Data Availability

The datasets generated and/or analysed during the current study are not publicly available due to privacy/ethical restrictions but are available from the corresponding author on reasonable request.

## Abbreviations

BIC: Bayes Information Criterion
BIRCH: Balanced Iterative Reducing and Clustering using Hierarchies
CFQ 11: Chalder Fatigue Scale
HFMPS-SF: Multidimensional Perfectionism Scale – short form
IPAQ-SF: Physical Activity Questionnaire – short form
ICD: International Classification of Diseases
K10: Kessler Psychological Distress Scale
LSP: Both shoulders pain – left concentrated
MSK: Musculoskeletal
NMQ: Nordic Musculoskeletal Questionnaire
OO: Other-oriented (sub-scale score HFMPS-SF)
PAS – QuickDASH: Performing Arts Section of the Quick Disabilities of the Arm, Shoulder and Hand Outcome Measure
PDI: Pain Disability Index
PRMDs: Playing-related musculoskeletal disorders
PSEQ-2: 2-item short form of the Pain Self-efficacy Questionnaire
RISMUS: Risk of Music Students study
RSP: Right shoulder pain
SO: Self-oriented (sub-scale score HFMPS-SF)
SP: Socially prescribed (sub-scale score HFMPS-SF)
SRH: Self-Related Health
VAS: Visual Analogue Scale
WHO: World Health Organization
WP: Wrist pain
WSP: Widespread pain
