# Correction to: Patterns of pain location in music students: a cluster analysis

**DOI:** 10.1186/s12891-021-04141-8

**Published:** 2021-03-10

**Authors:** Cinzia Cruder, Marco Barbero, Emiliano Soldini, Nigel Gleeson

**Affiliations:** 1 Department of Business Economics, Rehabilitation Research Laboratory 2rLab, Health and Social Care, University of Applied Sciences and Arts of Southern Switzerland, Manno/Landquart, Switzerland; 2 Centre for Health, Activity and Rehabilitation Research, Queen Margaret University, Edinburgh, UK; 3 Department of Research and Development, Conservatory of Southern Switzerland, Lugano, Switzerland; 4 Department of Business, Research Methodology Competence Centre, Health and Social Care, University of Applied Sciences and Arts of Southern Switzerland (SUPSI), Manno, Switzerland

**Correction to: BMC Musculoskelet Disord 22, 184 (2021)**

**https://doi.org/10.1186/s12891-021-04046-6**

Following the publication of the original article [1] an error was introduced into Fig. 1 during production, such that the numbers of participants reporting MSK pain in each anatomical area were not visible. The article has now been updated and the correct version of the figure is included in the online PDF and HTML.

**Fig. 1:**
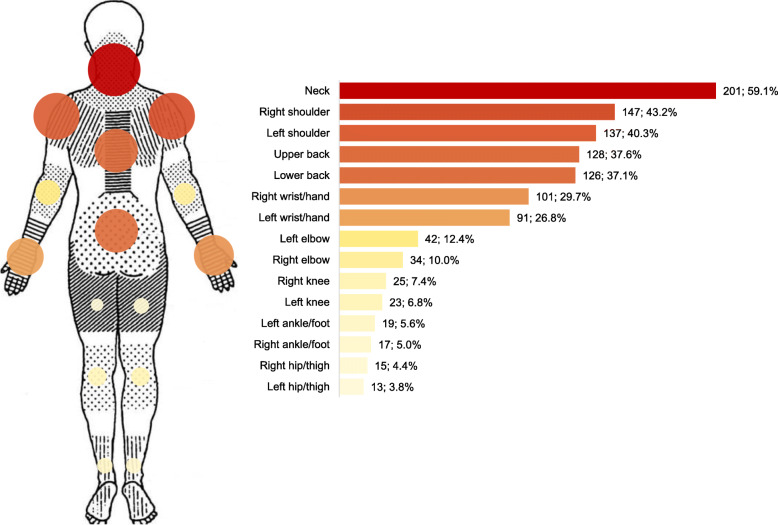
. Distribution of MSK pain location among participants. The anatomical areas and the layout of the original Nordic Questionnaire [24] with the affected areas, as well as the graph with the number of participants who self-reported MSK pain in specific areas of the body, have been reported. Dark red represents the most frequently reported area throughout all participants

The publisher apologises for this error.
